# In this month’s Bulletin 

**DOI:** 10.2471/BLT.23.000823

**Published:** 2023-08-01

In the editorial section, Susanna Hausmann-Muela (494) describes the Botnar Foundation’s view of WHO’s urban health research agenda.

Gary Humphreys (497 – 498) reports on the incentives needed to support antibacterial innovation. Maria Asuncion Silvestre talks to Gary Humphreys (499 – 500) about exclusive breastfeeding as part of neonatal care.

## Angola, Benin, Burkina Faso, Cameroon, Central African Republic, Chad, Côte d’Ivoire, Democratic Republic of the Congo, Equatorial Guinea, Gabon, Ghana, Guinea, Guinea-Bissau, Liberia, Mali, Niger, Nigeria, Republic of the Congo, Senegal, Sierra Leone, South Sudan, Togo

### Eliminating African trypanosomiasis 

Gerardo Priotto et al. (522 – 528), (529 – 534), (535 – 540), (541 – 545), (546 – 548) present four target product profiles of diagnostic tests.

## Indonesia

### Health insurance and contraception 

Asri Maharani et al. (513 – 521) study associations between coverage and access in a national family planning census.

## Global

### National action plans for antimicrobial resistance 

Scott JC Pallett et al. (501 – 512) analyse data variability across surveillance platforms.

